# Supplementary material for: A UMLS-based spell checker for natural language processing in vaccine safety
Source: BMC Med Inform Decis Mak. 2007 Feb 12;7:3. doi: 10.1186/1472-6947-7-3 (PMC1805499; doi:10.1186/1472-6947-7-3)
Supplement: Additional file 13 — RAP application directory. Sets up directory for RAP (RDF application for PHP) source code [file 1472-6947-7-3-S13.gz › rap/doc/phpdoc/dataset/IteratorFindQuadsMem.html]

xml version="1.0" encoding="iso-8859-1"?


Docs For Class IteratorFindQuadsMem


## Class IteratorFindQuadsMem

Description

Description |
Methods (details)

Implementation of a quad iterator.

This Iterator should be used like: for($iterator = $dataset->findInNamedGraphs(null,null,null,null); $iterator->valid(); $iterator->next()) { $currentQuad=$it->current(); };

History: <LI>05-11-2005 : First version of this class.</LI>

- access: public
- author: Daniel Westphal (http://d-westphal.de)
- version: V0.9.1

Located in /dataset/IteratorFindQuadsMem.php (line 30)

Method Summary

Description |
Methods (details)

IteratorFindQuadsMem
IteratorFindQuadsMem
(Resource $subject, Resource $predicate, Resource $object, dataset &$graphIterator, [Boolean $returnAsTriples = false])

mixed
current
()

integer
key
()

void
next
()

void
rewind
()

boolean
valid
()

Methods

Description |
Methods (details)

Constructor IteratorFindQuadsMem (line 118)

Constructor.

$subject, $predicate, and $object are used like find(). $getSPO supports the strings 's', 'p', and 'o' to return either the subject, predicate, or object of the result statements.

- access: public

IteratorFindQuadsMem

IteratorFindQuadsMem
(Resource $subject, Resource $predicate, Resource $object, dataset &$graphIterator, [Boolean $returnAsTriples = false])

- Resource
  $subject
- Resource
  $predicate
- Resource
  $object
- dataset
  &$graphIterator
- Boolean
  $returnAsTriples

current (line 193)

Returns the current item

- access: public

mixed

current
()

key (line 207)

Returns the key of the current item

- access: public

integer

key
()

next (line 157)

Moves Iterator to the next item in the list

- access: public

void

next
()

rewind (line 133)

Resets iterator list to start

- access: public

void

rewind
()

valid (line 147)

Says if there are additional items left in the list

- access: public

boolean

valid
()

Documentation generated on Thu, 7 Jul 2005 13:41:49 +0200 by phpDocumentor 1.3.0RC3
